# Supplementary material for: Hot spot prediction in protein-protein interactions by an ensemble system
Source: BMC Syst Biol. 2018 Dec 31;12(Suppl 9):132. doi: 10.1186/s12918-018-0665-8 (PMC6311905; doi:10.1186/s12918-018-0665-8)
Supplement: Supplementary file 2 — All relevant MATLAB code. A simple Matlab implement of our predictor. (ZIP 19,742 kb) [file 12918_2018_665_MOESM2_ESM.zip › Additional file 2/Read ASA/ASA Property/ASEdb/1DVFA.txt]

 NetSurfP - Protein Surface Accessibility and Secondary Structure Predictions

  

|  |  |  |
| --- | --- | --- |
|  |  | NetSurfP - Protein Surface Accessibility and Secondary Structure Predictions  Technical University of Denmark |

  


---

```
# For publication of results, please cite:
# A generic method for assignment of reliability scores applied to solvent accessibility predictions.
# Bent Petersen, Thomas Nordahl Petersen, Pernille Andersen, Morten Nielsen and Claus Lundegaard
# BMC Structural Biology 2009, 9:51 doi:10.1186/1472-6807-9-51
#
# Column 1: Class assignment - B for buried or E for Exposed - Threshold: 25% exposure, but not based on RSA
# Column 2: Amino acid
# Column 3: Sequence name
# Column 4: Amino acid number
# Column 5: Relative Surface Accessibility - RSA
# Column 6: Absolute Surface Accessibility
# Column 7: Z-fit score for RSA prediction
# Column 8: Probability for Alpha-Helix
# Column 9: Probability for Beta-strand
# Column 10: Probability for Coil
E D  1DVFA                  1    0.800 115.294   0.483   0.003   0.003   0.994
B I  1DVFA                  2    0.282  52.133  -0.570   0.004   0.514   0.481
E V  1DVFA                  3    0.450  69.196   0.877   0.002   0.816   0.182
B L  1DVFA                  4    0.152  27.904   0.535   0.002   0.816   0.182
E T  1DVFA                  5    0.439  60.903   0.353   0.003   0.718   0.279
B Q  1DVFA                  6    0.138  24.611   0.017   0.004   0.420   0.576
E S  1DVFA                  7    0.425  49.822   0.064   0.004   0.085   0.910
E P  1DVFA                  8    0.466  66.182  -1.014   0.018   0.019   0.964
E A  1DVFA                  9    0.519  57.238  -1.303   0.053   0.043   0.903
E S  1DVFA                 10    0.368  43.176  -1.300   0.022   0.359   0.619
B L  1DVFA                 11    0.229  41.948  -0.047   0.021   0.756   0.223
E S  1DVFA                 12    0.403  47.278  -0.668   0.018   0.846   0.136
B A  1DVFA                 13    0.235  25.952  -0.158   0.021   0.756   0.223
E S  1DVFA                 14    0.389  45.532   0.292   0.004   0.420   0.576
E V  1DVFA                 15    0.519  79.832  -0.020   0.004   0.085   0.910
E G  1DVFA                 16    0.639  50.266  -1.385   0.005   0.045   0.951
E E  1DVFA                 17    0.385  67.242   0.319   0.004   0.138   0.858
E T  1DVFA                 18    0.536  74.343   0.993   0.003   0.718   0.279
B V  1DVFA                 19    0.078  11.989   0.361   0.001   0.959   0.040
E T  1DVFA                 20    0.400  55.480   1.267   0.001   0.959   0.040
B I  1DVFA                 21    0.052   9.638   0.356   0.001   0.959   0.040
E T  1DVFA                 22    0.356  49.377   0.618   0.001   0.900   0.099
B C  1DVFA                 23    0.027   3.735   0.274   0.002   0.816   0.182
E R  1DVFA                 24    0.428  98.035   1.176   0.002   0.816   0.182
B A  1DVFA                 25    0.258  28.487  -1.226   0.003   0.718   0.279
B S  1DVFA                 26    0.342  40.036  -1.540   0.004   0.420   0.576
E G  1DVFA                 27    0.542  42.671  -2.448   0.004   0.085   0.910
E N  1DVFA                 28    0.441  64.592  -2.439   0.018   0.047   0.935
E I  1DVFA                 29    0.450  83.250  -1.994   0.018   0.088   0.893
E H  1DVFA                 30    0.358  65.157  -1.342   0.019   0.141   0.840
B N  1DVFA                 31    0.234  34.243  -0.765   0.005   0.336   0.660
B Y  1DVFA                 32    0.097  20.622   0.669   0.003   0.718   0.279
B L  1DVFA                 33    0.030   5.530   0.070   0.018   0.846   0.136
B A  1DVFA                 34    0.070   7.681   0.362   0.011   0.918   0.071
B W  1DVFA                 35    0.032   7.672   0.085   0.018   0.846   0.136
B Y  1DVFA                 36    0.042   9.061   0.224   0.018   0.846   0.136
B Q  1DVFA                 37    0.140  24.933   0.551   0.021   0.756   0.223
B Q  1DVFA                 38    0.087  15.467   0.244   0.021   0.451   0.528
E K  1DVFA                 39    0.403  82.938   1.464   0.004   0.085   0.910
E Q  1DVFA                 40    0.376  67.225   0.024   0.005   0.015   0.979
E G  1DVFA                 41    0.777  61.111  -1.998   0.003   0.003   0.994
E K  1DVFA                 42    0.496 101.965  -0.174   0.005   0.015   0.979
E S  1DVFA                 43    0.384  45.017  -0.066   0.018   0.047   0.935
B P  1DVFA                 44    0.104  14.743   0.192   0.005   0.262   0.733
B Q  1DVFA                 45    0.239  42.596   0.353   0.002   0.816   0.182
B L  1DVFA                 46    0.095  17.394   0.618   0.001   0.900   0.099
B L  1DVFA                 47    0.027   5.035   0.842   0.001   0.959   0.040
B V  1DVFA                 48    0.020   3.089   0.711   0.001   0.959   0.040
B Y  1DVFA                 49    0.089  19.041   0.065   0.001   0.959   0.040
B Y  1DVFA                 50    0.100  21.413  -0.416   0.001   0.900   0.099
B T  1DVFA                 51    0.159  22.026  -0.349   0.004   0.514   0.481
B T  1DVFA                 52    0.215  29.821  -0.759   0.004   0.197   0.799
E T  1DVFA                 53    0.564  78.157  -1.213   0.004   0.085   0.910
E L  1DVFA                 54    0.401  73.441   0.018   0.018   0.047   0.935
E A  1DVFA                 55    0.572  63.089  -1.686   0.018   0.047   0.935
E D  1DVFA                 56    0.722 104.098  -1.299   0.018   0.047   0.935
E G  1DVFA                 57    0.600  47.196  -1.408   0.018   0.047   0.935
E V  1DVFA                 58    0.289  44.419   0.183   0.018   0.047   0.935
E P  1DVFA                 59    0.602  85.481  -0.359   0.004   0.085   0.910
E S  1DVFA                 60    0.390  45.731  -1.725   0.004   0.197   0.799
B R  1DVFA                 61    0.261  59.861  -0.157   0.004   0.616   0.381
B F  1DVFA                 62    0.050  10.015  -0.444   0.002   0.816   0.182
B S  1DVFA                 63    0.217  25.397  -0.308   0.001   0.900   0.099
B G  1DVFA                 64    0.146  11.498  -2.309   0.001   0.900   0.099
E S  1DVFA                 65    0.357  41.829   0.234   0.002   0.816   0.182
E G  1DVFA                 66    0.427  33.629  -0.479   0.004   0.514   0.481
E S  1DVFA                 67    0.582  68.187  -1.562   0.004   0.138   0.858
E G  1DVFA                 68    0.686  53.965  -0.893   0.005   0.045   0.951
E T  1DVFA                 69    0.418  57.921  -0.476   0.004   0.197   0.799
E Q  1DVFA                 70    0.420  74.994   0.734   0.003   0.718   0.279
B Y  1DVFA                 71    0.068  14.553   0.053   0.001   0.900   0.099
B S  1DVFA                 72    0.256  29.956   0.993   0.001   0.900   0.099
B L  1DVFA                 73    0.031   5.676   0.377   0.001   0.959   0.040
E K  1DVFA                 74    0.284  58.398   0.921   0.001   0.900   0.099
B I  1DVFA                 75    0.072  13.394   0.015   0.002   0.816   0.182
E N  1DVFA                 76    0.442  64.738   0.314   0.004   0.514   0.481
E S  1DVFA                 77    0.494  57.932  -0.407   0.005   0.262   0.733
B L  1DVFA                 78    0.117  21.496   0.179   0.004   0.138   0.858
E Q  1DVFA                 79    0.459  81.924   0.788   0.004   0.085   0.910
E P  1DVFA                 80    0.449  63.770  -0.540   0.018   0.088   0.893
E E  1DVFA                 81    0.547  95.578  -0.771   0.053   0.043   0.903
B D  1DVFA                 82    0.221  31.846  -0.651   0.052   0.084   0.864
B F  1DVFA                 83    0.234  46.984   0.039   0.021   0.279   0.699
B G  1DVFA                 84    0.052   4.108  -0.213   0.021   0.756   0.223
B S  1DVFA                 85    0.161  18.869   0.814   0.018   0.846   0.136
B Y  1DVFA                 86    0.038   8.056   0.610   0.018   0.846   0.136
B Y  1DVFA                 87    0.057  12.181   1.148   0.018   0.846   0.136
B C  1DVFA                 88    0.020   2.836   1.154   0.021   0.756   0.223
B Q  1DVFA                 89    0.037   6.662  -0.497   0.021   0.756   0.223
B H  1DVFA                 90    0.112  20.355  -0.114   0.022   0.552   0.426
B F  1DVFA                 91    0.237  47.506  -0.531   0.021   0.279   0.699
E W  1DVFA                 92    0.486 116.907  -1.517   0.018   0.088   0.893
E S  1DVFA                 93    0.501  58.764  -2.358   0.018   0.047   0.935
E T  1DVFA                 94    0.388  53.760  -1.312   0.018   0.088   0.893
E P  1DVFA                 95    0.410  58.236  -0.293   0.020   0.205   0.775
B R  1DVFA                 96    0.255  58.372   0.062   0.021   0.451   0.528
B T  1DVFA                 97    0.143  19.862  -1.340   0.022   0.552   0.426
B F  1DVFA                 98    0.121  24.265  -0.812   0.004   0.514   0.481
B G  1DVFA                 99    0.118   9.255  -2.646   0.005   0.262   0.733
E G  1DVFA                100    0.422  33.180  -0.591   0.004   0.085   0.910
B G  1DVFA                101    0.269  21.178  -1.979   0.004   0.197   0.799
B T  1DVFA                102    0.149  20.611  -0.174   0.004   0.514   0.481
E K  1DVFA                103    0.418  85.983   1.478   0.001   0.900   0.099
B L  1DVFA                104    0.092  16.864   0.367   0.001   0.959   0.040
B E  1DVFA                105    0.272  47.449   0.910   0.001   0.959   0.040
B I  1DVFA                106    0.109  20.220  -0.259   0.001   0.959   0.040
E K  1DVFA                107    0.505 103.940   1.414   0.001   0.900   0.099
E R  1DVFA                108    0.663 151.827  -0.040   0.005   0.045   0.951

Explain the output.  Go back.
```

---
